# Sensorized Motor and Cognitive Dual Task Framework for Dementia Diagnosis: Preliminary Insights From a Cross-Sectional Study

**DOI:** 10.2196/64255

**Published:** 2025-10-06

**Authors:** Gianmaria Mancioppi, Erika Rovini, Laura Fiorini, Radia Zeghari, Auriane Gros, Valeria Manera, Philippe Robert, Filippo Cavallo

**Affiliations:** 1 Department of Industrial Engineering Faculty of Biomedical Engineering University of Florence Firenze Italy; 2 Cognition Behaviour Technology Laboratory Université Côte d'Azur Nice France; 3 Jean Louis Noisiez Fondation Biot France

**Keywords:** dementia, mild cognitive impairment, digital biomarkers, motor and cognitive dual task, early diagnosis

## Abstract

**Background:**

This study explores the use of novel motor and cognitive dual task (MCDT) approaches, based on upper limb motor function (ULMF) and lower limb motor function (LLMF), to discern individuals with mild cognitive impairment (MCI) or subjective cognitive impairment (SCI) from older adults who are cognitively healthy (OA).

**Objective:**

The study objectives encompass (1) the exploration of alternatives to the traditional walking MCDT; (2) the examination of various ULMF and LLMF MCDT modalities, incorporating different exercises with varying motor difficulties; and eventually, (3) the assessment of OA in comparison with people with MCI and SCI to acquire more nuanced insights into different stages of the diseases.

**Methods:**

The upper and lower limb motor performances of 44 older adults were evaluated using a wearable inertial system during 5 MCDTs comprising 2 ULMF tasks (forefinger tapping [FTAP] and thumb-forefinger tapping [THFF]) and 2 LLMF tasks (toe tapping heel pin [TTHP] and heel tapping toe pin [HTTP]). The gold standard for MCDT, 10-meter walking (GAIT), was included. We incorporated 5 pooled indices based on MCDTs, demographic data, and clinical scores into logistic regression models.

**Results:**

In 2-class classification models (MCI vs OA), HTTP showed the highest accuracy, at 93%; TTHP and TTHF models reached 89% accuracy; and FTAP and GAIT achieved 85% accuracy in distinguishing between the 2 groups of participants. In 3-class classification models (MCI vs SCI vs OA), transitioning from FTAP to THFF improved participant characterization by +5%. TTHP outperformed HTTP by +9%. Furthermore, models effectively identified individuals with MCI, with HTTP achieving 76% recall and TTHP achieving 88% recall.

**Conclusions:**

This study emphasizes the potential of an integrated, sensorized MCDT framework that combines a broader theoretical foundation and task selection with neuropsychological and behavioral data. This approach can enhance our understanding of dementia and provide clinicians with valuable diagnostic tools. Although these tasks demonstrated ease and efficiency, validation in subsequent clinical studies is necessary.

## Introduction

### Background

The aging global population has led to a surge in age-related neurodegenerative diseases, with Alzheimer disease (AD) affecting 46.6 million people worldwide. The lack of effective treatments is partly attributed to the inclusion of patients in the late stages of disease in studies. Therefore, the current approach to dementia, particularly AD, focuses on enhancing early identification and prognosis by identifying robust and trustworthy biomarkers [1]. The scientific community is searching for reliable, affordable, and noninvasive indicators that can be seamlessly integrated into clinical settings. The recent widespread availability of digital devices plays a central role in providing the opportunity to introduce new digital biomarkers [2,3]. The underlying brain pathology for most types of dementia develops over decades before cognitive symptoms emerge [4]. To address this issue, it has been recommended to assess motor function in addition to standard neuropsychological assessments. There is strong evidence that this type of assessment can aid in detecting mild cognitive impairment (MCI) or the risk of dementia in older adults [5]. In this regard, gait has been the most studied motor biomarker, with robust evidence showing that cognitive impairment is associated with gait impairments [6]. In the field of motor biomarkers, a promising approach is the motor and cognitive dual task (MCDT) paradigm [7]. This method leverages the motor-cognitive interference effect, suggesting that movements require cognitive resources [8]. Performing a motor task (generally walking) simultaneously with a cognitive one (counting backwards or performing a naming task) can overload neural networks, leading to atypical motor performance and revealing subtle cognitive impairment [9-11]. The MCDT approach is particularly promising in the clinical setting because it is easy to deploy and uncovers motor abnormalities, exacerbating subtle atypical motor performances [10]. Moreover, the MCDT can be implemented through various technologies, such as electronic walkways, optical systems, portable devices, and wearable sensors [7]. Therefore, this assessment can rely on objective measures and produce the advocated digital motor biomarkers.

### Prior Work

Among the studies using this approach, the overwhelming majority used walking MCDT. For instance, a walking MCDT protocol has been used to differentiate between older adults who are cognitively healthy (OA), individuals with MCI, and individuals with full-blown AD [12]. Notably, this study, along with [13] and [14], stands out as one of the few in which kinematic parameters were collected using inertial measurement units (IMUs). In contrast, the impact of various cognitive tasks performed by OA and individuals with MCI or AD has also been investigated [15]. In this study, the authors used an electronic walkway, the most commonly used electronic system for measuring gait parameters. Furthermore, several other notable studies used the aforementioned technological solution. Particularly, they endeavored to distinguish between OA and people presenting with amnestic and nonamnestic MCI, investigating their performance in MCDT involving counting backwards by 7 and naming animals [16]. In another noteworthy study, the potential predictive value of the MCDT approach was demonstrated, distinguishing individuals with MCI who would progress to AD from those who would not [17]. Importantly, in [18] and [19], the authors also attempted to differentiate between OA and individuals with MCI not only using walking MCDT but also by adopting an MCDT based on upper limb functions. This method, called upper limb motor function (ULMF) described in [20], offers an alternative to the walking task in MCDT protocols. Although the neurocognitive mechanisms underlying ULMF MCDT changes are not yet fully understood, it appears likely that they are comparable to those for gait [20]. Assessing ULMF may offer additional benefits since many subtle gait measures are imperceptible through clinical observation and necessitate cumbersome and expensive electronic gait analysis systems, limiting widespread access [21,22]. ULMF analysis is generally more accessible, and tests can be performed while seated and even at home. Emerging evidence indicates that various ULMF changes may contribute to discrimination from healthy aging [18,19,23,24]. Nonetheless, the specific tasks of ULMF to be adopted, the optimal measurement methods, and the movement variables associated with cognitive impairment remain unclear. A significant gap in the current literature has been highlighted, emphasizing the lack of consistency in the experimental methods used to assess ULMF [20]. Moreover, these studies have compared OAs with only one group of individuals with clinically manifested cognitive impairment, in particular AD. The trajectory of ULMF changes across the dementia continuum remains unclear. Authors suggest that future research should aim to recruit participants at earlier stages of dementia pathology, including nosographic categories such as subjective cognitive impairment (SCI), MCI, and early-stage dementia, to provide richer insights into changes related to disease progression. In this study, we addressed the aforementioned inquiries and sought answers to the following questions: (1) Which MCDT protocol is superior for identifying cognitive decline? and (2) Can comparing OA with those in the early stages of cognitive decline offer more comprehensive insights into changes associated with disease progression? To address these questions, we described and compared the performance of 5 MCDTs: the 10-meter walking task, considered the gold standard for MCDT (referred to as GAIT), and 4 tapping tasks—2 finger-tapping tasks (forefinger tapping [FTAP] and thumb-forefinger tapping [THFF]) and 2 foot-tapping tasks (toe tapping [TTHP] and heel tapping [HTTP]). The inclusion of foot-tapping tasks represents an innovation in MCDT protocols. In this work, we expanded our protocol to include 2 tasks related to lower limb motor function (LLMF). This decision was based on our prior work [23,24], where we compared the efficacy of different MCDT tasks, including finger- and foot-tapping tasks, alongside the gold standard GAIT MCDT. Moreover, in another work [25], we delved into the use of LLMF tasks by comparing different MCDT protocols based on 3 foot-tapping tasks. The rationale behind considering options beyond ULMF is rooted in the reality of working with older individuals who may have limitations in movement capabilities and range of motion (eg, hand pathologies such as rheumatoid arthritis and carpal tunnel syndrome). A diverse range of exercises enhances the clinician’s ability to assess the cognitive status of the participant. Moreover, our previous work [23,24] demonstrated that the MCDT based on foot-tapping tasks outperformed MCDT encompassing a walking task or ULMF for identifying OA from those with MCI and SCI. In this study, we present the participants’ performance from a kinematic perspective, and all motor parameters were obtained using wearable technology for upper and lower limb motion analysis [26,27]. Our objective was to differentiate OA from individuals with MCI and SCI. Following the process described in [28], we derived synthetic indices, termed the pooled THFF, from kinematic parameters extracted during MCDTs. This approach involves merging source variables from different domains and with distinct scoring ranges into a single summary score [28,29]. By creating new meaningful scores that complement existing neurological and neuropsychological tools, we aimed to build upon the findings of previous studies [7]. This proposal expands the array of motor tasks that can be included in an MCDT protocol, culminating in the development of an innovative decision support tool for physicians.

### Goal of This Study

Our objectives encompassed (1) the exploration of alternatives to the traditional walking MCDT, with a specific emphasis on MCDT protocols grounded in ULMF and LLMF; (2) the examination of various ULMF and LLMF MCDT modalities, incorporating different exercises with varying motor difficulties and adhering to standardized procedures, aiming to delineate a taxonomy of ULMF and LLMF MCDT protocols for identifying the condition of MCI; and eventually, (3) the assessment of OA in comparison with individuals with MCI and SCI to acquire more nuanced insights into the progression of the respective conditions.

## Methods

### Recruitment

This study was conducted within the framework of the Marco-Sens multicentric project. A total of 44 participants were enrolled for this investigation. Each participant underwent a series of clinical evaluations, including blood tests, encephalic magnetic resonance imaging, and neuropsychological assessments. These assessments comprised the Mini-Mental State Examination (MMSE), recognized as the gold standard for dementia screening; Free and Cued Selective Reminding Test (FCSRT), designed to evaluate long-term verbal memory capabilities; Digit Span Test, in both forward and backward forms, to assess short-term verbal memory and working memory; verbal fluency test in its semantic category version, aimed at evaluating linguistic repertoire and mental flexibility; Trail Making Test (TMT) form A, which assesses selective attention; and TMT form B, which evaluates working memory abilities. Among the 44 participants enrolled, 17 (39%) were classified as having MCI by the clinician from the Marco-Sens study. In particular, the criteria adopted for the diagnosis of MCI were the following: (1) concern regarding a change in cognition from the patient, a knowledgeable informant, or a skilled clinician observing the patient; (2) objective evidence of impairment (from cognitive testing) in one or more cognitive domains; (3) preservation of independence in functional abilities (although individuals may be less efficient and make more errors than in the past); and (4) no evidence of a significant impairment in social or occupational functioning (ie, “not demented”) [30]. On the other hand, 17 of the 44 (39%) recruited participants were classified by the clinicians of the Marco-Sens study as individuals with SCI, by adopting the following diagnostic criteria: (1) presence of subjective cognitive deficits; (2) belief that one’s cognitive capacities have declined in comparison with 5 years or 10 years previously; (3) absence of significant medical, neurologic, or psychiatric conditions, including depression and anxiety disorders, that might interfere with cognition; (4) absence of overt cognitive deficits (elicited in the context of a detailed clinical interview or evident to a spouse or other informants); (5) cognitive performance in the general normal range; and (6) absence of dementia. [31]. Eventually, 10 (10/44, 22%) were identified as OA. All the participants were recruited and evaluated at the Memory Center of Nice University Hospitals (Nice, France) and the Université Cote d’Azur’s CoBTeK research lab.

In this cross-sectional study, we excluded participants presenting with (1) sensory or motor impairments, (2) moderate to severe cognitive impairment (MMSE<24 adjusted for age and educational level), and (3) problems understanding questions or counting numbers as well as individuals who had (4) participated in any cognitive stimulation or training program. In addition, all participants completed the Frontal Assessment Battery (FAB) for research purposes.

### Ethical Considerations

The study was conducted in accordance with the Helsinki Declaration and complied with both institutional guidelines and French national regulations on human research. It was approved by the French Research Ethics Committee - Comité de Protection des Personnes on April 15, 2019 (number ID RCB: 2019-A00342-55). All participants were given thorough written explanations of the study’s objectives and procedures. Before participating, they provided written informed consent. All personal data were pseudonymized at the source and stored on local devices with restricted access. The data were then securely transferred to the CoBTeK server, accessible only by authorized personnel. Participants were volunteers, and no compensation was provided.

### Instrumentation

**Figure 1:**
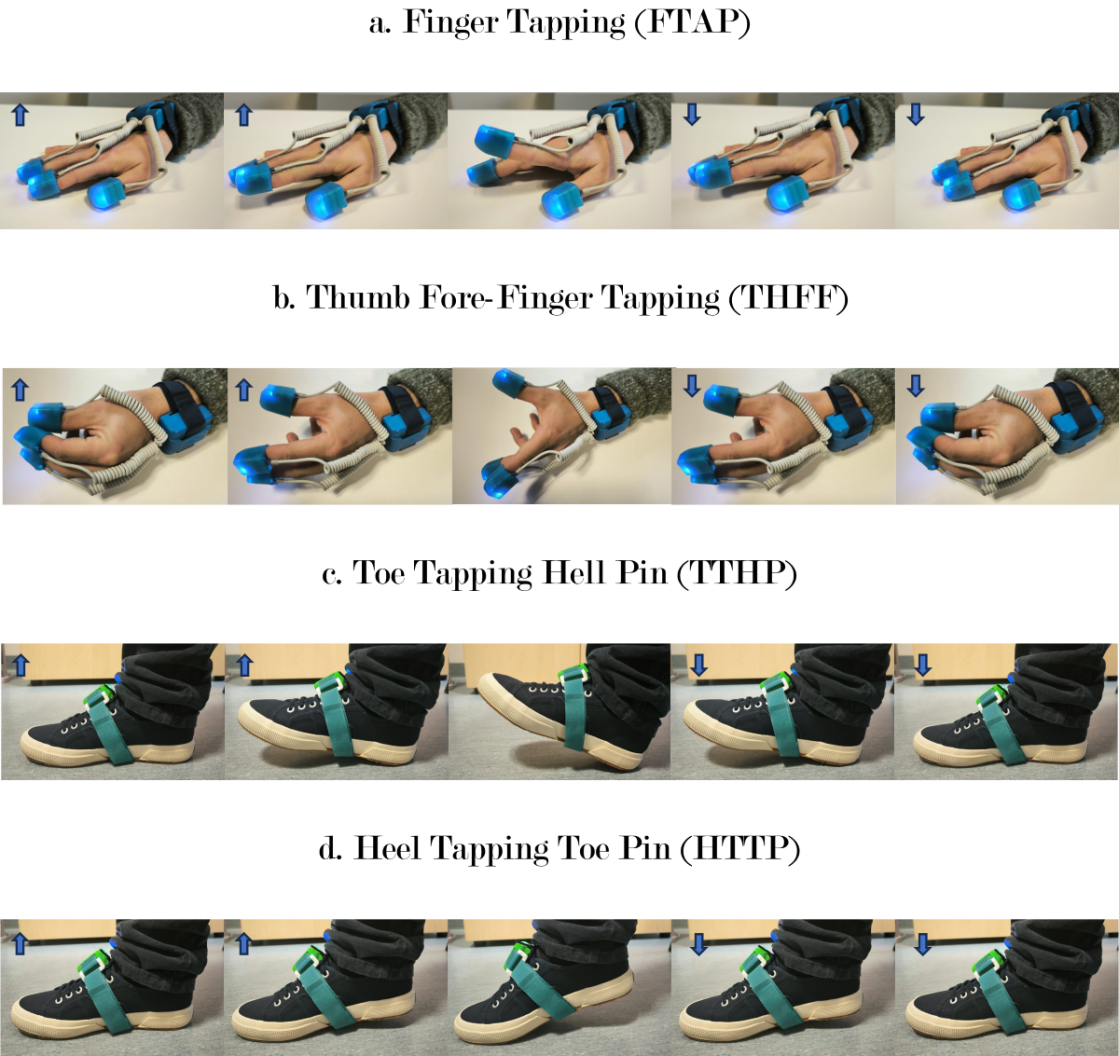
Upper and lower motor and cognitive dual task protocols, with blue arrows representing the movement direction: (A) forefinger tapping (FTAP), (B) thumb-forefinger tapping (THFF), (C) toe tapping heel pin (TTHP), and (D) heel tapping toe pin (HTTP).

We used a wearable system with microelectromechanical sensors (MEMS) divided into 2 modules: 1 for upper limb motor assessment and 1 for lower limb motor assessment [26]. The system included 4 wearable devices based on IMUs (2 SensHand and 2 SensFoot) to collect and objectively analyze participants’ motor performance during the experimental test. The system is low cost, low power, noninvasive, small, lightweight, wireless, and easy to use. The 2 SensHand devices were used for the motion analysis of the upper limbs [32]. Each one is composed of 4 modules, equipped with an iNEMO-M1 board with dedicated STM32F103RE microcontrollers (ARM 32-bit Cortex-M3 CPU, STMicroelectronics), and based on MEMS that include a 3-axis digital gyroscope L3G4200D (STMicroelectronics) and an STMicroelectronics 6-axis geomagnetic module LSM303DLHC. The module placed on the wrist acts as the coordinator of the system and is also equipped with a ParaniTM-ESD210 (Sena Technologies Inc) Bluetooth serial device for wireless communication to a control station. The other sensors are placed on the distal phalanges of the thumb, index finger, and middle finger and included in silicon finger stalls printed with different sizes to be adapted for each participant. Module coordination and data synchronization is implemented through the controller area network (CAN-bus) standard (see Figures 1A and 1B). For the analysis of the lower limbs, 2 SensFoot sensors were used instead [33]. Each consists of an IMU integrated into the iNEMO-M1 board based on MEMS (L3G4200D 3-axis gyroscope and LSM303DLHC 6-axis geomagnetic module) and an STM32F103RE ARM-based 32-bit microcontroller (STMicroelectronics). The system is integrated with an SPBT2632C2A Bluetooth module (v.3.0, STMicroelectronics) that wirelessly transmits acquired data to a remote personal computer for offline analysis. The device is placed on the dorsum of the participant’s foot using a Velcro strap to ensure no movements occur between the foot and the sensor (see Figures 1C and 1D). Both the sensors for the feet and the coordinators of the hand sensors are included in a plastic cover created using a 3D printing technique. Data were collected on a PC through a custom-made interface developed in Visual Studio, C# language. For this study, the sampling frequency was set to 100 Hz. Bluetooth modules allowed for the wireless transmission of acquired data to a remote computer, and a graphical user interface enabled clinicians to save data and analyze motion parameters offline [32].

### Experimental Protocol

This study included 5 MCDTs, 2 of which concerned ULMF (FTAP and THFF), while the others concerned LLMF (TTHP and HTTP) [33]. The final task was the MCDT benchmark of walking ability (GAIT).

Each exercise was carried out as a single motor task (ST) and under 3 distinct dual task (DT) conditions with different levels of cognitive difficulties. The ST condition refers to a motor exercise with no cognitive load (CL0), whereas the DT condition includes 3 CL levels (CL1, CL2, CL3, namely, counting backwards by 1, 3, and 7, respectively) [23]. Counting backwards was chosen as the cognitive task [7] because it represents a popular neuropsychological test and is a cognitive task that it is relatively simple to modify by requesting greater mental manipulation, involving a larger numerical sequence, and demanding increased attention and concentration [34]. In Table 1, the entire list of tasks encompassed in the experimental protocol is shown, and specific information regarding task procedures, sensor placement, and duration is provided. Notably, each participant performed the task in a random order to avoid systematic effects of learning or fatigue.

**Table 1:** Completed schema of the experimental protocol.

Tasks			Description		Single-task condition		Dual-task conditions
**Upper limb motor function**							
	FTAP^a^	Participants must be (1) still, with their hand lying on the table, for 3 seconds, then they (2) started tapping their forefinger, at their own pace, for 15 seconds. In the meantime, their forearm rests on the experimental desk.		CL^b^0: The participant performs only the motor task.		CL1: perform the task while counting backwards by 1; CL2: perform the task while counting backwards by 3; CL3: perform the task while counting backwards by 7	
	THFF^c^	Participants must be (1) still, with their hand lying on the table, for 3 seconds, then they (2) started tapping their thumb and their forefinger, at their own pace, for 15 seconds. In the meantime, their forearm rests on the experimental desk.		CL0: The participant performs only the motor task.		CL1: perform the task while counting backwards by 1; CL2: perform the task while counting backwards by 3; CL3: perform the task while counting backwards by 7	
**Lower limb motor function**							
	TTHP^d^	Participants had to (1) keep their foot still on the ground for 3 seconds, then they (2) started tapping their toe, at their own pace, for 15 seconds while their heel stayed on the floor.		CL0: The participant performs only the motor task.		CL1: perform the task while counting backwards by 1; CL2: perform the task while counting backwards by 3; CL3: perform the task while counting backwards by 7	
	HTTP^e^	Participants had to (1) keep their foot still on the ground for 3 seconds, then they (2) started tapping their heel, at their own pace, for 15 seconds while their toe stayed on the floor.		CL0: The participant performs only the motor task.		CL1: perform the task while counting backwards by 1; CL2: perform the task while counting backwards by 3; CL3: perform the task while counting backwards by 7	
**Walking task**							
	GAIT^f^	Participants had to (1) stand for 3 seconds, then they (2) started walking straight for 10 meters, followed by (3) staying still for 3 seconds.		CL0: The participant performs only the motor task.		CL1: perform the task while counting backwards by 1; CL2: perform the task while counting backwards by 3; CL3: perform the task while counting backwards by 7	

^a^FTAP: forefinger tapping.

^b^CL: cognitive load.

^c^THFF: thumb-forefinger tapping.

^d^TTHP: toe tapping heel pin

^e^HTTP: heel tapping toe pin

^f^GAIT: 10-meter walking

Figure 1 encompasses the innovative tasks included in the MCDT protocol. The experimenter instructed the participants to complete each task at their own pace, with no guidance on task prioritization (physical task vs counting task).

### Signal Processing and Feature Extraction

Accelerations and angular velocities acquired by the system were stored on a PC during the acquisitions and processed offline using Matlab (The MathWorks Inc).

#### Preprocessing

All signals were filtered using a fourth-order low-pass digital Butterworth filter. For the protocols based on ULMF and LLMF, the cutoff frequency was set to 5 Hz to process acceleration and angular rate data, effectively removing high-frequency noise. In contrast, for gait analysis, the cutoff frequency was set to 3 Hz, as the gait cycle involves slower movements than the more rapid tapping actions [33].

#### Signal Segmentation and Event Detection

For each movement exercise, the most informative signals were identified for processing. Concerning finger tapping, for FTAP and THFF, the angular velocity of the index finger that was orthogonal to the plane where the tapping movement occurred was selected (ie, ωy or angular velocity around the y axis). Concerning the foot-tapping exercises (TTHP and HTTP), the angular velocity orthogonal to the movement was chosen (ie, ωy). Eventually, GAIT was analyzed in the sagittal plane (ie, considering αx, αz, and ωy), which is the direction of the motion. The selected signals were then segmented, identifying characteristic times that delimit typical patterns in the signal according to the type of task. For the exercises FTAP, THFF, TTHP, and HTTP, 3 characteristic times were defined (ie, when the action started, when the movement reached the maximum amplitude, and when the action ended); thus, the movements were divided into 2 phases: opening phase and closing phase. Differently, for gait analysis, the typical gait cycle phases were identified according to [32], resulting in identifying 4 characteristic times (ie, when the foot started to move, when the toe lifted off the ground, when the heel struck the ground, and when the foot was completely flat) and the related static and swing phases. A detailed description of the methods for signal segmentation and event detection for each task is reported in Multimedia Appendix 1.

#### Feature Extraction

Subsequently, motor parameters were extracted, as described in [27] and [23]. In particular, angular rates selected and segmented during the previous step were integrated to calculate the amplitude of the movements using the trapezoidal rule, with subintervals of integration equal to 100 ms, which is the inverse of the sensor-sampling rate. Linear drift correction was applied step by step to avoid cumulative effects, according to the theory of the zero velocity update. Thus, the correction at each step (eg, each finger tap, each step while walking) allowed us to restrain the accumulation of errors. Based on the characteristic times, 32 features were extracted from FTAP, TTHP, THFF, and HTTP (8 each). Eventually, 13 parameters were extracted from the GAIT task (see Table 2). We extracted a total of 45 parameters, calculated for each CL. Thus, 4 datasets were estimated (CL0, CL1, CL2, CL3). By the end of this process, a total of 180 features were computed and analyzed.

All the formulas for the parameters extracted are reported and described in Multimedia Appendix 1.

**Table 2:** Complete list and description of kinematic parameters extracted and adopted in motor and cognitive dual task (MCDT) protocols.

Tasks and their sequence		Parameter	Definition	Unit of measure
**FTAP^a^, THFF^b^, TTHP^c^, and HTTP^d^**				
	1	Tap	Number of taps	[ ]
	2	exc	Excursion	degrees
	3	excSD	Excursion SD	degrees
	4	wo	Opening velocity	degrees/s
	5	woSD	Opening velocity SD	degrees/s
	6	wc	Closing velocity	degrees/s
	7	wcSD	Closing velocity SD	degrees/s
	8	IAV	Energy expenditure	m/s
**GAIT^e^**				
	1	GT	Gait time	s
	2	GSTRD	Gait stride	[ ]
	3	GVEL	Gait velocity	m/s
	4	GSTRD-L	Gait stride length	m
	5	GSTRD-T	Gait stride time	s
	6	GSTRD-T-SD	Gait stride time SD	s
	7	GSWT	Gait swing time	s
	8	GSWT-SD	Gait swing time SD	s
	9	GSTT	Gait stance time	s
	10	GSTT-SD	Gait stance time SD	s
	11	GRS	Gait relative stance	[ ]
	12	GEXC	Gait excursion	degrees
	13	GEXC-SD	Gait excursion SD	degrees

^a^FTAP: forefinger tapping.

^b^THFF: thumb-forefinger tapping.

^c^TTHP: toe tapping heel pin.

^d^HTTP: heel tapping toe pin.

^e^GAIT: 10-meter walking.

### A Weighted DT Cost

The DT cost (DTC) is the difference “in cost” between a feature extracted during the MCDT and its baseline value. The results of this subtraction are divided by the ST value and multiplied by 100 (see equation 1):



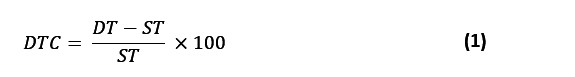



Standard DTC ignores some critical aspects of the cognitive counterpart of MCDT protocols. It normalizes the participant’s response to their motor baseline without taking into account their cognitive performance. We identified 2 factors related to cognitive performance: (1) cognitive efficiency (the participant’s ability to execute the cognitive task) and (2) commitment (how engaged they were). These 2 parameters can have an impact on the final DTC calculation. In recent work by Mancioppi et al [24,25], we used the number of correct responses given by the participant during the tasks as a proxy for cognitive efficiency and commitment. As a result, we compute a weighted version of DTC that is the product of the kinematic parameters of interest and the number of correct cognitive answers standardized on our sample (see equation 2).



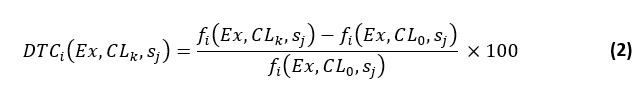



The general notation for feature is *f*_i_ (*Ex*,*CL*_k_,*s*_j_), Ɐ *i* ꓱ{0,...45}, Ɐ *k* ꓱ{0,...3}, Ɐ *j* ꓱ{0,...44} where *i* is the *i*-th feature computed (as described in Table 2) and *j* is the *j*-th participant performing the task, while *Ex* indicates the executed exercises (FTAP, THFF, TTHP, HTTP, GAIT) and *CL*_k_ (where *k*=0, 1, 2, 3) represents the CL level as described in the previous paragraphs.

This technique makes it possible to determine the percentage changes in participants’ performance on MCDTs. However, we believe that the DTC, as computed so far, normalizes the participant’s response regarding their motor baseline without acknowledging their cognitive performance. To overcome this limitation, we identified 2 factors related to the participant’s ability to perform a cognitive task: (1) cognitive efficiency and (2) participant’s commitment. Misleading results could be produced by not considering these data. For instance, if a participant does not count while performing the task, it could be that the participant is not able to perform the cognitive task (their cognitive efficiency is too low) or the participant is not willing to perform the task (their commitment is too low). This situation can represent a limit case in which the obtained values at *f*_i_(*Ex,CL*_k_*,s*_j_) and *f*_i_(*Ex,CL*_0_*,s*_j_)are basically equivalent; therefore, the DTC calculated would be near 0%, and the performance would be considered normal.

We identified the number of correct responses (see Multimedia Appendix 1), referred to as *Nc*(*Ex*, *CL*_k_, *s*_j_), as a proxy for cognitive efficiency and commitment. For example, an elevated number of generic responses (both correct and incorrect) tells us that the participant is properly engaged by the task and cooperating but does not tell us about cognitive efficiency (ie, there is a high number of responses but a low accuracy). Conversely, a high response accuracy tells us that the participant can perform the task, but, for example, the participant may stop counting after a couple of subtractions because the task is too difficult and they do not want to fail in front of the experimenter or because they are not willing to carry out the task (high accuracy but low commitment). On the other hand, a high number of correct responses tells us that the participant is properly engaged by the task and that they are committed but also tells us something about the participant’s cognitive efficiency.

Thus, we computed an adjusted version of each *f*_i_(*Ex*, *CL*_k_, *s*_j_). It represents the product of *f*_i_(*Ex*, *CL*_k_, *s*_j_)and the respective *Z*-scored *Nc*(*Ex*, *CL*_k_, *s*_j_), which is referred to as *Z*_c_(*Ex*, *CL*_k_, *s*_j_). Clearly, this calculation is possible only where *k*>0; therefore, it is not possible at baseline (see equation 3):



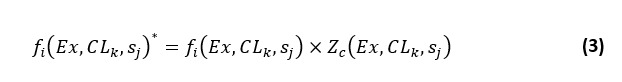



Notably, we used a particular equation (see equation 4) that forces the distribution to be between 0.01 (to avoid 0 values) and 1. Note that “a” and “b” refer to the minimum (0.01) and maximum values, respectively:



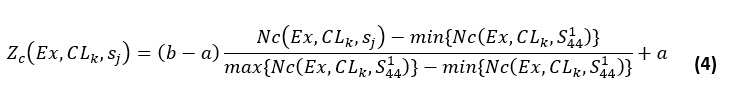



We used this weighted *f*_i_*f*_i_(*Ex*, *CL*_k_, *s*_j_)*value instead of the standard one in the *DTC*_i_(*Ex*, *CL*_k_, *s*_j_)calculation. Hereinafter, we will refer to the *DTC*_i_(*Ex*, *CL*_k_, *s*_j_) values calculated using *f*_i_*f*_i_(*Ex*, *CL*_k_, *s*_j_)* instead of the general *f*_i_(*Ex*, *CL*_k_, *s*_j_) as *DTC*_i_(*Ex*, *CL*_k_, *s*_j_)*.

### Pooled Index Development

Due to the small sample size, we used a feature reduction strategy that aggregates the weighted DTC within each exercise of our MDCT into 5 pooled indices. We calculated 5 pooled indices for the MCDT exercises. These pooled indices were fed into a classification model aiming to predict participants’ diagnostic categories. It is recommended that each pooled index includes up to 6 components with low pairwise correlations in order to optimize the pooled index’s ability to convey information. It is thus possible to cover important measurement domains while reducing the variability of final pooled index scores [29].

**Figure 2:**
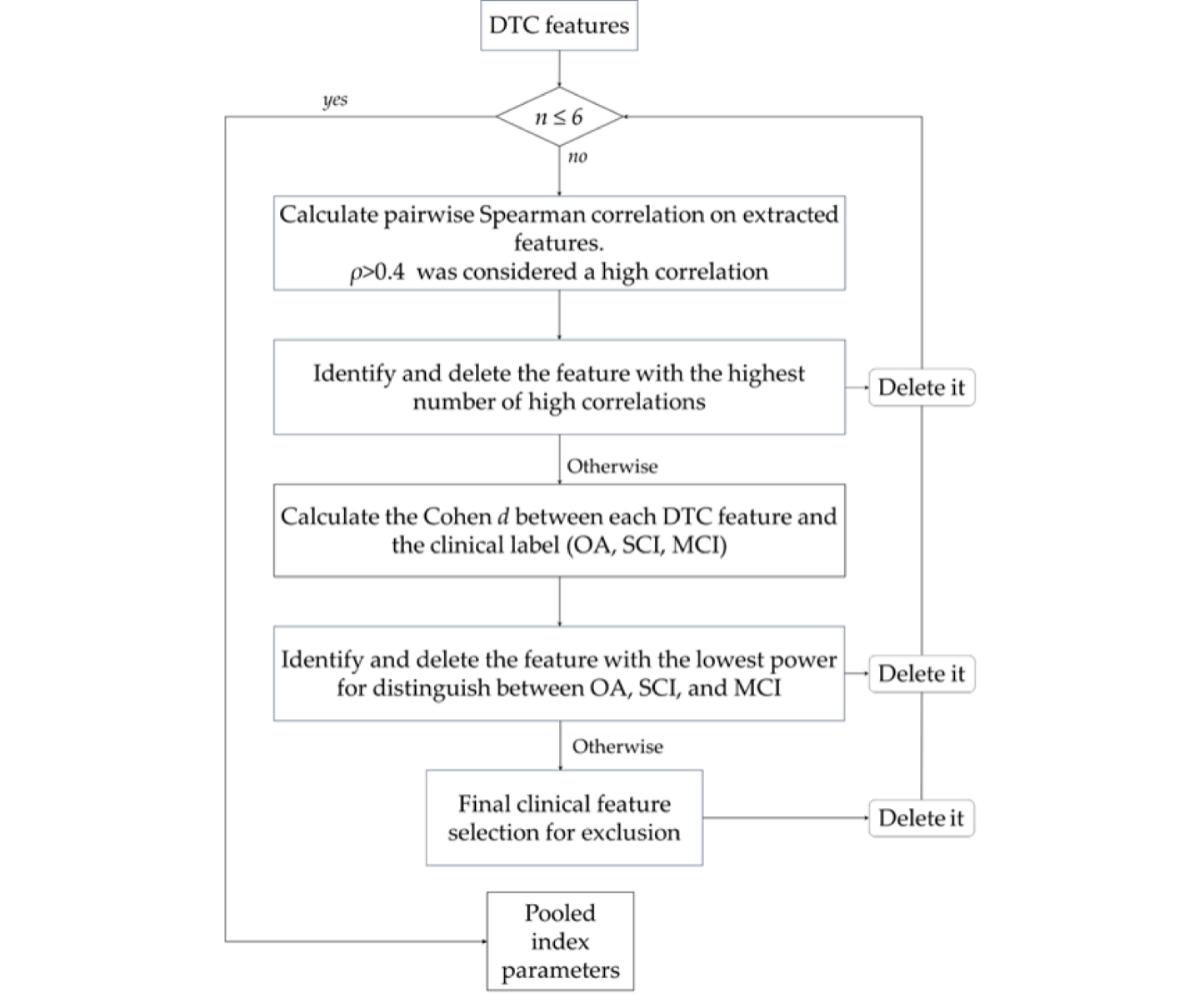
Feature selection process, with the criteria summarized in the diamond-shaped blocks. DTC: dual task cost; MCI: mild cognitive impairment; OA: older adults who are cognitively healthy; SCI: subjective cognitive impairment.

To define up to 6 features for each pooled index, we followed these 3 steps: First, we performed a pairwise Spearman correlation among the *DTCs*^∗^ features to select the most unrelated parameters and perform a first features screening. A threshold was fixed at *rho*<0.4. The number of *rho* above the threshold was counted for each feature. The feature that presented the highest number of *rho* above the threshold was deleted. Second, when such a criterion was not applicable (2 or more features present the same, and highest, number of *rho* above the threshold), we deleted the variable that presented the lower effect size (computed using Cohen *d*) on the diagnosis. Third, when both step 1 and step 2 outputs were not able to select the parameters to be discarded, theoretical considerations were used to establish which one to keep and which one to drop. Following this approach, we selected up to 6 *DTCs*^∗^ parameters to build the pooled indices. Importantly, all the variables were recorded, so that higher values indicated a greater level of functioning. That is crucial since the pooled indices were obtained by calculating a normalized score for each parameter selected then averaging those scores in one final pooled index for each participant. See Figure 2 for an overview of the pooled index development.

### Statistical Analysis

Preliminary descriptive analyses of demographic and cognitive data for OA and individuals with SCI or MCI were conducted. The chi-square test was used to assess differences in gender and educational level among the 3 groups. Because the normality and homoskedasticity assumptions were violated (as determined using the 1-sample Kolmogorov-Smirnov test and Engle autoregressive conditional heteroskedasticity test), Kruskal-Wallis tests were used to highlight age differences among groups. To determine where the difference occurred, post hoc Mann-Whitney tests (with Bonferroni correction) were included. In addition, we used pooled index scores in multivariate logistic regression models to assess their ability to solve classification problems among discrete outputs (individual diagnoses: OA, SCI, MCI). Our classification models were tested on 2 classes (OA vs MCI) and 3 classes (OA vs SCI vs MCI). Models include pooled indices, age, and FAB test scores. We used age to control for the effect of age across groups. As a result, we included FAB scores to add a pure cognitive dimension to our model. The results of a multivariate logistic model were validated using a leave-one-out cross-validation method, which involves iteratively using a single observation from the dataset as the test set while the remaining observations form the training set, ensuring that each observation is used exactly once as the test set. We described our models’ performance by calculating sensitivity, specificity, and accuracy (for the 2-class model), as well as recall, precision, *F*_1_-score, and overall accuracy (for the 3-class models). Multimedia Appendix 1 contains information about the formula for the evaluation metrics we adopted. MATLAB (version 2018b) was used to perform all analyses.

## Results

This section outlines the results of our investigation into the impact of various motor loads on MCDT performance. We commence by providing an analysis of the sociodemographic and neuropsychological characteristics of our sample. Subsequently, we present the findings of our study using 3- and 2-group classification models based on 5 MCDT metrics. The classification models presented here were designed to differentiate between 2 ULMF MDCT (FTAP and THFF) and 2 LLMF MCDT (TTHP and HTTP) based on motor performance metrics acquired using the SensHand and SensFoot instruments [26,27].

### Sociodemographic Data and Neuropsychological Data

A total of 44 older adults were enrolled in our study, comprising 26 women (59%) and 18 men (41%). The participants had a median age of 69.5 (IQR 13.5) years. Of the participants, 17 were diagnosed with MCI, and 17 were diagnosed with SCI. The remaining 10 participants were classified as OA. Significant differences were found between the age of the OA group and those of the SCI and MCI groups (Kruskal-Wallis test: *P*=.02; post hoc Mann-Whitney tests: OA vs SCI, *P*=.01, and OA vs MCI, *P*=.01; critical *P* value with Bonferroni correction *P*=.02). No significant age differences were observed between the groups of individuals with SCI and MCI. Statistically significant differences were also found in the FAB scores among all 3 groups (Kruskal-Wallis test: *P*=.02; post hoc Mann-Whitney tests: OA vs SCI, *P*=.03, OA vs MCI, *P*<.001, and SCI vs MCI, *P*=.002; critical *P* value with Bonferroni correction: *P*=.02). Refer to Table 3 for the sociodemographic data and clinical information.

**Table 3:** Demographic and neuropsychological variables of the 3 groups.

Variables		Total sample (N=44)	OA^a^ (n=10)	SCI^b^ (n=17)	MCI^c^ (n=17)	Statistics (*df*)		*P* value	
Female, n (%)		26 (59)	6 (60)	7 (41)	13 (76)	4.38 (2)^d^		.11	
Age (years), median (IQR)		69.5 (13.5)	63 (9)	72 (13.5)	73 (12.75)	8.32 (43)^e^		*.*02^f^	
**Education level, n (%)**							4.44 (4)^d^		.35
	Primary	4 (9)	0	1 (6)	3 (18)				
	Secondary	12 (27)	2 (20)	4 (23)	6 (35)				
	Superior	28 (64)	8 (80)	12 (71)	8 (47)				

^a^OA: older adults who are cognitively healthy.

^b^SCI: subjective cognitive impairment.

^c^MCI: mild cognitive impairment.

^d^Chi-square test.

^e^Kruskal-Wallis test.

^f^Mann-Whitney post-hoc tests (with Bonferroni correction) comparing pairs of groups resulted in significant differences between OA and SCI and between OA and MCI.

### Logistics Regression Model Results

#### Overview

To enhance the robustness of our logistic regression models and isolate the effect of motor-cognitive interference when distinguishing among our groups of participants, we integrated demographic and neuropsychological variables with MCDT pooled index values to control for their potential effects. Specifically, we incorporated age as a covariate, considering its established association with MCI and dementia. This adjustment also helps address slight imbalances in sample sizes across different participant classes. Additionally, we included FAB scores as a measure of executive cognitive function, given the relationship between the cognitive task encompassed in the MCDT and executive functioning, such as working memory and selective attention [34]. As a result, we constructed a total of 5 logistic regression models, including 2 models for ULMF, FTAP, and THFF and 2 models for LLMF, TTHP, and HTTP. We evaluated the performance of all models against the MCDT gold standard GAIT.

#### 2-Class Classification Models (MCI vs OA)

Models incorporating FTAP or GAIT performance indices achieved comparable accuracy in differentiating between individuals with MCI and OA, with an accuracy of 85%. However, classification models based on TTHP and THFF outperformed these models, achieving an accuracy of 89% (+4%). The model based on HTTP achieved the highest accuracy rates, reaching 93% (+8%). In conclusion, THFF and LLMF models surpassed the gold standard GAIT by +4%, +4%, and +8%, respectively, while FTAP performed the same as GAIT. Moreover, THFF achieved +4% when compared with FTAP, and HTTP achieved +4% when compared with TTHP (see Table 4).

**Table 4:** Cross-validated results of logistic regression models for identifying older adults who are cognitively health (OA) versus mild cognitive impairment (MCI) as well as OA versus subjective cognitive impairment (SCI) versus MCI using demographics (age) and neuropsychological results (Frontal Assessment Battery [FAB] scores) in combination with various pooled indices.

Regressors			OA vs MCI							OA vs SCI vs MCI							OA *F*_1_-score, %			SCI *F*_1_-score, %			MCI *F*_1_-score, %
			Sensitivity, %		Specificity, %		Accuracy, %		Recall^a^, %			Precision^a^, %		*F*_1_-score^a^, %									
**Upper limb motor function**																							
	FTAP^b^, age, FAB^c^ [24]	88		80		85		59			59		59		56			53			67		
	THFF^d^, age, FAB	88		82		89		64			64		64		70			57			64		
**Lower limb motor function**																							
	TTHP^e^, age, FAB [24]	88		82		89		77			77		77		74			71			86		
	HTTP^f^, age, FAB	94		90		93		68			68		68		63			63			76		
**GAIT^g^**																							
	GAIT, age, FAB [24]	88		80		85		66			66		66		60			61			74		

^a^Weighted averages of those parameters.

^b^FTAP: forefinger tapping.

^c^FAB: Frontal Assessment Battery.

^d^THFF: thumb-forefinger tapping.

^e^TTHP: toe tapping heel pin.

^f^HTTP: heel tapping toe pin.

^g^GAIT: 10-meter walking.

#### 3-Class Classification Models (MCI vs OA vs SCI)

**Figure 3:**
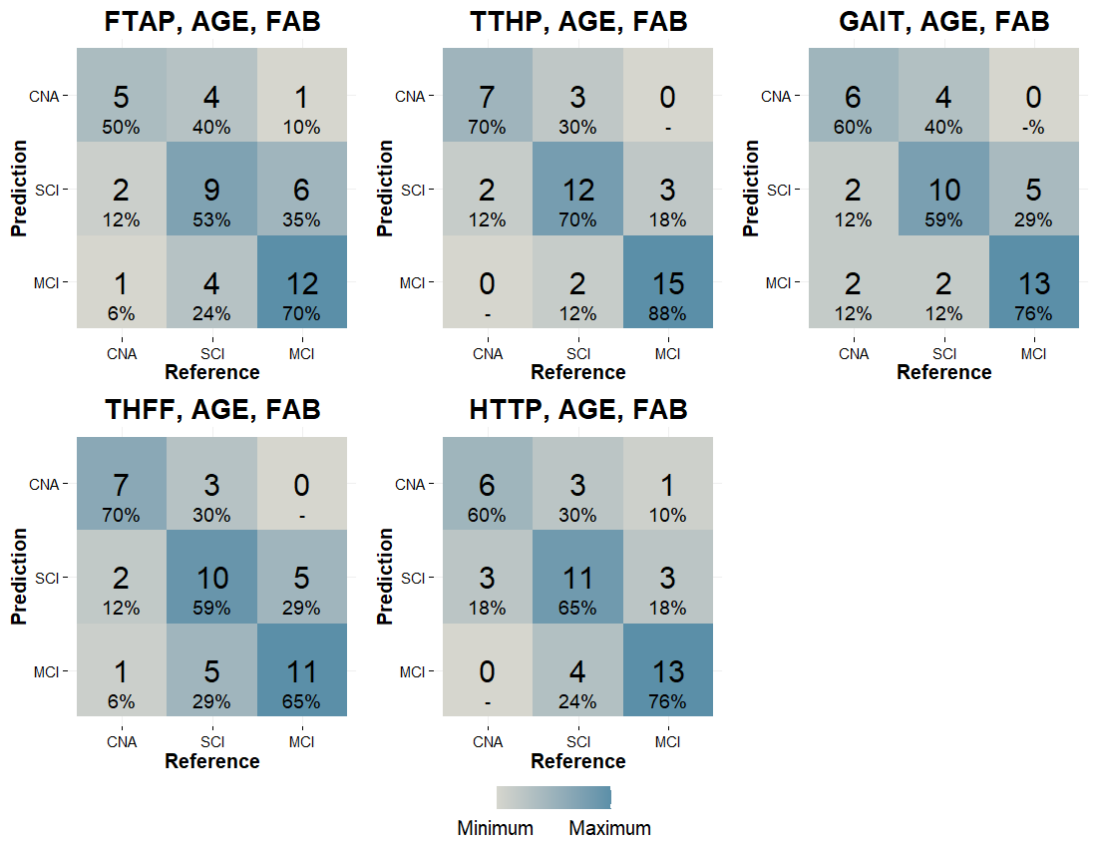
Confusion matrices of classification model performances, with the percentage scores representing the performance of the model on the prediction rows: (A) forefinger tapping (FTAP), age, Frontal Assessment Battery (FAB); (B) toe tapping heel pin (TTHP), age, FAB; (C) 10-meter walking gait test (GAIT), age, FAB; (D) thumb-forefinger tapping (THFF), age, FAB; and (E) heel tapping toe pin (HTTP), age, FAB.

In terms of the models’ capacity to differentiate among 3 groups of participants (SCI, MCI, and OA), they displayed distinct dynamics. Specifically, the models relying on ULMF performed similarly; the THFF model achieved a 5% increase in *F*_1_-score compared with the model constructed on FTAP. However, HTTP demonstrated lower performance than TTHP, with a discrepancy of –9%. Remarkably, the model developed using the gold standard metric (GAIT) attained an overall accuracy of 66% (see Table 4). Notably, Figure 3 shows that the model constructed with FTAP demonstrated satisfactory performance for identifying individuals with MCI, but its performance was lower for identifying OA and individuals with SCI. In contrast, the model based on THFF displayed better performance for identifying OA, thereby increasing the *F*_1_-score. However, the model constructed with TTHP demonstrated good performance for each group, particularly for MCI participants, while the model based on HTTP showed a decrease in performance, particularly for recognizing the OA and SCI groups. Figure 3 shows the confusion matrix of each classification model, particularly, for the multiclass classification problem (OA vs SCI vs MCI). The figure shows that the OA and MCI groups tended to not be confounded by the models. The maximum number of OA classified as MCI (or vice versa) was 2 participants, while the SCI group was generally misclassified: FTAP misclassified 8 SCI participants (4 as OA and 4 as MCI), 2 OA as SCI, and 6 MCI as SCI. THFF misclassified 8 SCI participants (3 as OA and 5 as MCI), 2 OA as SCI, and 5 MCI as SCI. TTHP misclassified 5 SCI participants (3 as OA and 2 as MCI), 2 OA as SCI, and 3 MCI as SCI. HTTP misclassified 7 SCI participants (3 as OA and 4 as MCI), 3 OA as SCI, and 3 MCI as SCI. GAIT misclassified 6 SCI participants (4 as OA and 2 as MCI), 2 OA as SCI, and 5 MCI as SCI.

The results indicate that various motor tasks can help distinguish between individuals with MCI and those without cognitive impairment. The limited cognitive resources of individuals with MCI are associated with poorer performance on MCDT, and different motor tasks, possibly reflecting varying motor-cognitive complexities, appear to amplify the differences between individuals with MCI and OA (2-class classification model). Notably, both the LLMF protocols and 1 of the 2 ULMF protocols (THFF) outpaced the performance of the gold standard (GAIT), while FTAP performed similarly to GAIT. These findings suggest that commonly used motor tasks can be incorporated into MCDT protocols and should be sufficiently complex to amplify the differences between individuals with MCI and OA. Similar to the aforementioned result, when distinguishing among SCI, MCI, and OA (3-class classification model), transitioning from an FTAP-based model to a THFF-based model appears to yield a significant improvement in participant characterization (+5%; see Table 4). On the other hand, the LLMF tasks altered the dynamics seen in the 2-class classification problem. In fact, in this multiclass classification problem, TTHP outperformed HTTP by +11% (see Table 4). Notably, for both ULMF and LLMF, all classifiers demonstrated sufficient accuracy for identifying individuals with MCI (into the 3-class classification problem). Specifically, the model built on HTTP achieved a recall degree of 76%, while the model built on TTHP achieved a recall degree of 88%. In contrast, the performance of the models for identifying OA and individuals with SCI was generally poorer (see Table 4). These results showed that the use of different ULMF and LLMF MCDT could lead to a higher accuracy for identifying individuals with MCI in the 2-class and 3-class classification problems. The formulas for all evaluation metrics (ie, sensitivity, specificity, accuracy, recall, precision, and *F*_1_-score) are provided in Multimedia Appendix 1.

## Discussion

### Principal Findings

This study aimed to assess the feasibility of designing, developing, and using innovative MCDT approaches for the screening and evaluation of individuals with MCI. In particular, the goal was to generate MCDT pooled indices for the effective differentiation between individuals with MCI and those without the condition (OA and people with SCI). Eventually, we aim to expand the literature on MCDT solutions and devise protocols that could enhance the clinical toolkit with a rapid and noninvasive method. To answer the aforementioned research goals, 4 logistic regression models were built: 2 models for ULMF (based on FTAP and THFF pooled indices) and 2 models for LLMF (based on TTHP and HTTP pooled indices). We evaluated the performance of all models against one based on MCDT gold standard (GAIT) pooled indices.

Each model achieved results equivalent to GAIT. Moreover, 3 of the 4 models (THFF, TTHP, HTTP) surpassed the GAIT performance (see Table 4). ULMF and LLMF MCDT exhibited different accuracies for identifying individuals with MCI compared with identifying OA. This suggests that they rely on similar mechanisms, as stated in [20], regarding gait and tasks based on upper limb functioning, although they are not perfectly overlapped. However, differences exist; regarding the ULMF MCDT models, THFF performed better than FTAP at identifying individuals with MCI against OA. This could imply that they engage the participants’ motor planning resources differently, possibly reflecting varying orders of motor complexity for each task. During the FTAP task, the participant is required to plan the movement and control just 1 finger, involving only 1 joint. In contrast, THFF asks the participant to articulate and coordinate 2 fingers, potentially increasing the level of motor complexity. This suggests that a higher order of motor complexity could widen the performance gap between OA and individuals with MCI. On the other hand, regarding the MCDT based on the lower limb tasks, even though one task performed better than the other (HTTP surpassed TTHP), the participants were required to control the same joint but recruit different muscles. Therefore, it is not as straightforward to identify different degrees of motor complexity in the 2 tasks, as was done for the tasks concerning the upper limbs. In this case, the differences in performance could still be attributed to slight variations in motor complexity from a motor program perspective, which should be explained in future work, or perhaps to differences in the automatization of the movement or even inherent characteristics of our sample.

On the contrary, attempting to distinguish individuals with MCI from OA and individuals with SCI represents a more complex task. Only the models based on the lower limb tasks (TTHP and HTTP) surpassed the gold standard GAIT, whereas the models based on upper limb tasks performed poorer than GAIT. Notably, regarding the ULMF models, it is possible to observe the same dynamic observed in the 2-class classification problem (THFF outperformed FTAP). Conversely, the models based on lower limb tasks did not show the same dynamic as observed in the 2-class classification problem, where HTTP performed better than TTHP. In this case, on the contrary, TTHP outpaced HTTP, and both remained superior to the GAIT model. Regarding the models based on upper limb motor tasks, the transition from FTAP to THFF, with a possible increased level of motor complexity, improved the model’s *F*_1_-score but did not meet the ability of the GAIT model to distinguish among the 3 classes of individuals. A more complex motor task might be needed to distinguish among 3 classes, which supports the results we obtained in previous work showing that the motor component of FTAP is too simple to be used in a multiclass classification problem [24]. In particular, as shown in Figure 3, the model built on FTAP performed poorly for identifying OA and individuals with SCI, while it worked better for individuals with MCI. Interestingly, THFF gained capabilities to identify OA more accurately than any other group, also increasing its ability to identify individuals with SCI, although its performance for recognizing individuals with MCI remained stable. Similarly, Figure 3 shows that, for the model based on FTAP, there was a low exchange of participants between OA and individuals with MCI (and vice versa), although this was a general tendency across all the models. In other words, the model mostly failed to recognize individuals with SCI, mistaking them for both OA and individuals with MCI. Notably, this phenomenon is still present in the model based on THFF but at a lower magnitude. This could possibly be explained by the fact that a simpler motor task is sufficient to differentiate between MCI and OA. In fact, the performance gap, determined by a higher motor cognitive interference in individuals with MCI, seems to be enough to identify them against a control group. That is no longer true if we approach a multiclass classification problem, including SCI participants. That could be explained by an inherent characteristic of SCI as a nosographic category; people diagnosed with SCI express concerns about their cognitive status without corroborating evidence from neuropsychological assessments. That means that their cognitive performance, although possibly lower than before, is still in the range of normality. Moreover, it is worth mentioning that a high percentage of individuals with SCI remain stable over the years without converting to MCI. These participants may show an association between their cognitive complaints and depression, anxiety, or personality traits, as well as other possible comorbidities. The percentage of people with SCI who develop MCI, showing an actual cognitive impairment, is between 5.6% and 18.9% [35]. Therefore, a simpler motor task seems to have low efficacy for distinguishing this group, while increasing the motor complexity and pushing the participants to work more on their cognitive limits allow the model to identify them better. For these reasons, it could be interesting to include the concept of motor complexity and define a degree of motor load for each motor task included in an MCDT protocol. Increasing the degree of motor load (as could happen in the passage from FTAP to THFF) of the ULMF MCDT would help to better define the groups or eventually reach a plateau. Nevertheless, addressing a multiclass classification problem requires a higher level of complexity, particularly if the group of SCI participants might encompass people who are cognitively unimpaired and who will not show cognitive decline in the future (similar to the OA group) and people who will actually develop MCI. Concerning the MCDT pooled indices based on LLMF tasks, they perform better at distinguishing individuals with MCI from OA and individuals with SCI, surpassing both the GAIT- and upper limb–based protocols. It is worth mentioning that TTHP obtains better results, particularly compared with HTTP for recognizing individuals with MCI. This contradicts the results obtained in the 2-class classification problem. Notably, in this case, it is not as simple as explaining the differences in model classification performance through the hypothesis of an increase in the motor load from one task to the other. Participants were required to articulate the same joint in 2 different ways. The possible increase in the motor complexity of the movement is less evident compared with the tasks of the upper limb. This ambiguity should be clarified, especially because the model based on HTTP is way better at identifying MCI versus OA in the 2-class classification problem, while it does not maintain the same characteristic in the multiclass classification problem. Again, it could be interesting to investigate the effect of motor tasks with different levels of motor difficulty to observe if it is possible to take advantage of this logic. A last point that is worth mentioning is that, concerning the multiclass classification problem, the *F*_1_-scores of the MCI class were always higher when compared with the other groups (see Figure 3). That could mean that pooled index MCDT-based models are particularly able to identify people with MCI. The only exception is THFF, which recognizes OA participants better. The hand task seems to be more sensitive when identifying OA than people with SCI or MCI maybe because of an inherent motor complexity and the requirement to program and organize fine hand movements instead of coarse large movements. Future work could study the combination of these models in a new model that could maintain the ability to identify individuals with MCI and increase the capability to recognize OA. In this study, the clinical data encompassed mainly executive functioning of the participants; that is, a strong relationship exists between MCDT performance and executive functioning ability, where neural correlates (eg, prefrontal cortex and dorsolateral prefrontal cortex) were activated [36,37]. It would be extremely interesting to take into consideration a wider neuropsychological battery that also considers memory, language, visuospatial abilities, and other behavioral aspects. In conclusion, a broader spectrum of MCDT protocols, encompassing both ULMF and LLMF tasks, appears to provide an available alternative to the traditional walking MCDT, yielding better results for identifying individuals with MCI and SCI in contrast to OA. In particular, LLMF achieved superior results in both the 2-class and 3-class classification problems. Although the examination of ULMF MCDT suggested that higher motor-cognitive interference (potentially due to an increased motor load between FTAP and THFF) could amplify differences between MCI and OA, as well as among MCI, SCI, and OA. The expansion of the MCDT repertoire would provide additional solutions in the clinical assessment of cognitive impairment arising from neurodegenerative diseases, enhancing clinical and diagnostic options while offering a deeper understanding of the progression of both motor and cognitive conditions. Specifically, the proposed set of MCDTs is intended to be executed using a single (IMU-based) wearable sensor system that can be used in several MCDT protocols, from standard walking tasks to fine hand movements. This technological approach offers a simple, cost-effective, and noninvasive option that can be seamlessly integrated into clinical practice, research labs, and potentially even in patients’ homes in the future. This aligns with the concept of a continuum of care, extending from hospitals to patients’ homes, facilitating early identification, timely intervention, and monitoring of therapy effects.

### Limitations

The findings of this investigation underscore the potential of a comprehensive sensorized MCDT framework that integrates innovative methods for calculating DTC with a broad task selection and that incorporates neuropsychological and behavioral data. Although these tasks hold promise for automated analysis and real-time assessment of patients, the results require validation in subsequent clinical studies. To address existing limitations, it is imperative to delve into the study of motor loads and their integration with diverse CLs. Identifying and examining additional levels of motor-cognitive interference, particularly those involving increased motor complexity, is essential to differentiate among OA, individuals with SCI, and individuals with MCI. Augmenting the sample size within the OA group could yield more robust results and elucidate certain dynamics while increasing the MCI group sample size and stratifying for subtypes of MCI could provide greater insight into this nosographic category.

### Comparison With Prior Work

A substantial and expanding body of literature addresses the study and quantification of motor-cognitive interference in individuals showing early stages of AD and in populations at high risk for dementia. However, the predominant approach, as highlighted in numerous journal articles and reviews [6,7,21,36,38], has focused primarily on gait analysis using MCDT approaches. Notably, a few authors have diverged from this trend, as articulated in a recent scoping review [20] that underscores the potential of studying ULMF instead of traditional walking tasks. The work [18,19] represents a robust approach to this topic. They propose that measures of motor function speed and accuracy within ULMF tests may provide adequate complexity for assessing cognitive impairment. Their studies demonstrated that counting backward by 3 within a ULMF MCDT experiment was sufficiently challenging to detect MCI in older adults. Additionally, they found that ULMF MCDT outperformed gait as the motor task component of the dual tasks. In alignment with this perspective, our previous research explored the introduction of alternative motor tasks within the dual-task framework, extending beyond the conventional walking task. Specifically, in [23,24], we examined the feasibility of incorporating both ULMF and LLMF tasks. This approach aimed to (1) evaluate the differences in motor control mechanisms between the upper and lower body, as well as their respective motor-cognitive interferences, and (2) provide viable alternatives for participants unable to walk or with limited upper limb mobility. Further, in [25], we explored various movements to be included in LLMF tasks, aiming to expand the MCDT framework and investigate the effects of different motor difficulties on motor-cognitive interference. Our prior work highlighted the potential of extending the MCDT protocol to include ULMF and LLMF tasks. In contrast, this study advances our previous efforts by incorporating a broader range of motor tasks and examining their differential efficacy for discerning among individuals with MCI, OA, and individuals with SCI. This study aimed to provide a more comprehensive framework for the application of MCDT approaches in clinical settings. We explored the potential effects of different motor difficulties on motor-cognitive interference, seeking to gather insights and delineate guidelines for the effective use of MCDT methodologies.

### Conclusions

This study emphasizes the potential of an integrated, sensorized MCDT framework that combines a broader theoretical foundation and task selection with neuropsychological and behavioral data to advance our understanding of dementia and equip clinicians with valuable tools. This approach aligns with the proposition of establishing a taxonomy of MCDT, as suggested in [38]. This differentiation is crucial as, although individuals with MCI can be relatively easily identified in contrast to OA or individuals with SCI using even low CLs, distinguishing between OA and individuals with SCI can be challenging. Therefore, higher CLs prove to be beneficial. An intriguing avenue for further exploration involves conducting a longitudinal study with individuals with SCI to observe if those initially identified as OA remain cognitively stable while those classified as MCI progress to develop cognitive impairment. Eventually, as our understanding of the relationship between motor-cognitive interference and cognitive decline deepens, it will be possible to refine and optimize clinical protocols, thereby better integrating them into hospital and clinical settings.

## Appendix

Multimedia Appendix 1 Formulas for the evaluation metrics.

## Abbreviations

AD: Alzheimer disease
CL: cognitive load
DT: dual task
DTC: dual task cost
FAB: Frontal Assessment Battery
FTAP: forefinger tapping
GAIT: 10-meter walking gait test
HTTP: heel tapping toe pin
IMU: inertial measurement unit
LLMF: lower limb motor function
MCDT: motor and cognitive dual task
MCI: mild cognitive impairment
MEMS: microelectromechanical sensors
OA: older adults who are cognitively healthy
SCI: subjective cognitive impairment
ST: single motor task
THFF: thumb-forefinger tapping
TMT: Trail Making Test
TTHP: toe tapping heel pin
ULMF: upper limb motor function
