# Supplementary material for: Sensorized Motor and Cognitive Dual Task Framework for Dementia Diagnosis: Preliminary Insights From a Cross-Sectional Study
Source: J Med Internet Res. 2025 Oct 6;27:e64255. doi: 10.2196/64255 (PMC12538184; doi:10.2196/64255)
Supplement: Multimedia Appendix 1 [file jmir_v27i1e64255_app1.docx]

A Sensorized Motor and Cognitive Dual-Task Framework for Dementia Diagnosis: Preliminary Insights from a Cross-Sectional StudySupplementary Material

Gianmaria Mancioppi^a^, Erika Rovini^b^, Laura Fiorini^a^, Radia Zeghari^b^, Auriane Gros^b^, Valeria Manera^b^, Philippe Robert^b^, Filippo Cavallo^a^

*aThe Department of Industrial Engineering, University of Florence, via Santa Marta 3, 50139, Florence, Italy.*

*bThe CoBTeK, Universite´ Cote d’Azur (UCA), 10 Rue Molie`re, 06100 Nice, France.*

# Introduction

The supplementary material encompasses the complete list and the description of 1) the signal processing procedure (encompassing the segmentation and event detection, and the feature extraction; 2) the tables encompassing the cognitive performance given by the subjects during the MCDT;3) Evaluation metrics description.

Notably, the red-highlighted reference within the text pertains to the bibliography of the manuscript.

# Signal Processing

## Segmentation and Events Detection

- FTAP
- T*_start_*: it starts the tapping when the forefinger tip moves away from the thumb finger. It is assumed that this transition occurs when $\omega_{y}\left( t \right)\geq TH_{FF}$ , where $TH_{FF}={15}^{\circ}/s$ is a threshold of the angular velocity to identify the beginning of the movement.
- T*_FF_*: the maximum amplitude of the movement has been reached and the forefinger and thumb are at the maximum distance. This transition occurs when $\omega_{y}\left( t \right)<0^{\circ}/s$ and the angular velocity changes its direction (*i.e.*, from clockwise to anticlockwise).
- T*_end_*: the movement is completed, and the forefinger tip contacts the thumb again. This transition occurs when $\omega_{y}\left( t \right)\geq TH_{FFv}$, where $TH_{FFv}=-3^{\circ}/s$ is an empiric threshold of the angular velocity to identify the index is stable again.
- THFF
- T*_start_*: it is the start of the tapping when the forefinger tip moves away from the thumb finger. It is assumed that this transition occurs when $\omega_{y}\left( t \right)\geq TH_{TF}$ , where $TH_{TF}={15}^{\circ}/s$ is a threshold of the angular velocity to identify the beginning of the movement.
- T*_TF_*: the maximum amplitude of the movement has been reached and the forefinger and thumb are at the maximum distance. This transition occurs when $\omega_{y}\left( t \right)<0^{\circ}/s$ and the angular velocity changes its direction (*i.e.*, from clockwise to anticlockwise).
- T*_end_*: the movement is completed, and the forefinger tip contacts the thumb again. This transition occurs when $\omega_{y}\left( t \right)\geq TH_{TFv}$ , where $TH_{TFv}=-3^{\circ}/s$ is an empiric threshold of the angular velocity to identify the index is stable again.
- TTHP
- T*_start_*: the tapping starts when the toe moves away from the ground. It is assumed that this transition occurs when $\omega_{y}\left( t \right)<TH_{TO}$, where $TH_{TO}={10}^{\circ}/s$ is a threshold of the angular velocity to identify the raising of the toe.
- T*_HS_*: only the heel gets in touch with the ground, and the toe reaches the highest position from the floor. This transition occurs when $\omega_{y}\left( t \right)<0^{\circ}/s$ and the angular velocity changes its direction (i.e., from clockwise to anticlockwise).
- T*_end_*: the movement is completed, and the foot is totally lying on the floor. This transition occurs when $\omega_{y}\left( t \right)>-TH_{FF}$, where $TH_{FF}=3^{\circ}/s$is an empiric threshold of the angular velocity to identify the foot flat phase.
- HTTP
- T*_start_*: the tapping starts when the heel moves away from the ground. It is assumed that this transition occurs when $\omega_{y}\left( t \right)<-TH_{HO}$, where $TH_{HO}={10}^{\circ}/s$ is a threshold of the angular velocity to identify the raising of the heel.
- T*_TO_*: only the toe gets in touch with the ground, and the heel reaches the highest position from the floor. This transition occurs when $\omega_{y}\left( t \right)>0^{\circ}/s$ and the angular velocity changes its direction (*i.e.* from anticlockwise to clockwise).
- T*_end_*: the movemen t is completed, and the foot is totally lying on the floor. This transition occurs when $\omega_{y}\left( t \right)<-TH_{FF}$, where $TH_{FF}=3^{\circ}/s$is an empiric threshold of the angular velocity to identify the foot flat phase.
- GAIT
- T*_start_*: it is the start of walking when the heel moves away from the ground. It is assumed that this transition occurs when $\omega_{y}\left( t \right)<-TH_{HO}$, where $TH_{HO}={50}^{\circ}/s$ is a threshold of the angular velocity to identify the raising of the heel from the ground.
- T*_TO_*: only the toe gets in touch with the ground. This transition occurs when $\omega_{y}\left( t \right)>0^{\circ}/s$, the toe is going to be off from the ground (i.e., toe-off time), and the angular velocity changes its direction (*i.e*., from anticlockwise to clockwise).
- T*_Hs_*: the heel gets in touch again with the ground (i.e., heel-strike time). This transition occurs when $\omega_{y}\left( t \right)<0^{\circ}/s$ and the angular velocity changes its direction (i.e., from clockwise to anticlockwise).
- T*_end_*: the movement is completed, and the foot is totally lying on the floor. This transition occurs when $\omega_{y}\left( t \right)>-TH_{FF}$, where $TH_{FF}=3^{\circ}/s$ is an empiric threshold of the angular velocity to identify the foot flat phase, *i.e.*, when the foot is stable on the ground

## Feature Extraction

This section includes the entire list of the parameters extracted from upper limb exercises (FTAP and THFF) and lower limb tasks (TTHP and HTTP), as well as the parameters extracted from the gold standard task (GAIT) (see Table 2).

- Number of tapping movements:

$$Taps = number of (T_{end})$$

- Mean of the maximum movement amplitude (i.e., the maximum angle reached by the toe in the TTHP, maximum angle reached by the heel during HTTP, the maximum angular distance between finger and thumb in THFF):

$$\text{Exc}=\frac{\sum_{i=2}^{\text{Taps}-1} \text{Exc}\left( i \right)}{\left( Taps-2 \right)}$$

Where:

$$\text{Exc}\left( i \right)=\max\left[ \int_{T_{\text{start}}}^{j} \omega_{y}\left( t \right)dt-\tilde{\theta}\left( j \right) \right]$$

with:

$$\tilde{\theta_{j}}=\left[ \frac{j-T_{\text{start}}}{T_{\text{end}}-T_{\text{start}}} \right]\theta T_{\text{end}}$$

- Standard deviation of the movement amplitude:

$$\text{ExcDs}=\sqrt{\frac{\sum_{i=2}^{\text{Taps}-1} \left( \text{Exc(i)}-\text{Exc} \right)^{2}}{\text{Taps}-3}}$$

- Mean of opening velocity overall movements:

$$\omega_{o}=\frac{\sum_{i=2}^{Taps-1} \omega_{o}\left( i \right)}{Taps-2}$$

where:

$$\omega_{o}=\text{mean }\left[ \omega_{y}\left( j \right) \right]$$

with:

$$j=T_{Start},\ldots,T_{TF}\left( or T_{FF},T_{HS},T_{TO} \right)$$

- Standard deviation of the movement opening velocity:

$$\omega_{o}SD=\sqrt{\frac{\sum_{i=2}^{\text{Taps}-1} \left( \omega_{o\text{(i)}}-\omega_{o} \right)^{2}}{\text{Taps}-3}}$$

- Mean of closing velocity overall movements

$$\omega_{c}=\frac{\sum_{i=2}^{Taps-1} \omega_{c}\left( i \right)}{Taps-2}$$

where:

$$\omega_{c}=\text{mean }\left[ \omega_{y}\left( j \right) \right]$$

With

$$j=T_{TF}+1\left( or T_{FF},T_{HS},T_{TO} \right),\ldots,T_{End}$$

- Standard deviation of the movement closing velocity:

$$\omega_{c}SD=\sqrt{\frac{\sum_{i=2}^{\text{Taps}-1} \left( \omega_{c\text{(i)}}-\omega_{c} \right)^{2}}{\text{Taps}-3}}$$

- Integral of the magnitude of the total acceleration vector (IAV), which is related to the estimated energy expenditure

$$IAV=\int\sqrt{\left( a_{x}^{2}+a_{y}^{2}+a_{z}^{2} \right)} dt$$

- Gait time to cover 10 meters:

$$\text{GT}=T_{\text{end}}end-T_{\text{start}}\left( 1 \right)$$

- Number of strides during 10 meters walking:

$$\text{GTSTRD}=\text{Number}\left( T_{\text{end}} \right)$$

- Gait Velocity:

$$\text{GVEL}=\frac{\text{spaced walked}}{\text{GT}}$$

- Mean Gait Stride Length:

$$\text{GSTRD-L}=\frac{\text{spaced walked}}{\text{GSTRD}}$$

- Mean Gait Stride Time:

$$\text{GSTRD-T}= \frac{\sum_{i=1}^{GTSTRD-1} T_{HS}(i+1)-\text{T}_{\text{HS}}\left( i \right)}{GTSTRD-1}$$

- Standard deviation of Gait Stride Time:

$$\text{GSTRD-T-SD}=\sqrt{\frac{\sum_{i=1}^{GTSTRD-1} \left( \text{GSTRD-T}\left( i \right)-\text{GSTRD-T} \right)^{2}}{\text{GSTRD-1}}}$$

- Mean Gait Swing Time:

$$\text{GSWT}=\frac{\sum_{i=1}^{GTSTRD-1} T_{HS}(i)-\text{T}_{\text{TO}}\left( i \right)}{\text{GTSTRD}}$$

- Standard deviation of Gait Swing Time:

$$\text{GSWT}=\sqrt{\frac{\sum_{i=1}^{\mathrm{GTSTRD}} \left( \text{GSWT}\left( i \right)-\text{GSWT} \right)^{2}}{\text{GTSTRD}}}$$

- Mean Gait Stance Time:

$$\text{GSTT}=\frac{\sum_{i=1}^{\mathrm{GTSTRD}} GSTRD-T\left( i \right)-GSWT-T\left( i \right)}{\text{GSTRD}}$$

- Standard deviation of Gait Stance Time:

$$\text{GSTT-SD}=\sqrt{\frac{\sum_{i=1}^{GTSTRD} \left( \text{GSTT}\left( i \right)-\text{GSTT} \right)^{2}}{\text{GTSTRD}}}$$

- Mean Relative Stance:

$$\text{GRS}=\frac{100\%}{\text{GSTRD}}\sum_{i=2}^{\text{GTSTRD}} \frac{\text{GSTT}\left( i \right)}{\text{GSTRD-T}\left( i \right)}$$

- Mean of the maximum dorsiflexion angular excursion of the foot over all the strides:

$$GEXC=\frac{\sum_{i=2}^{\text{GSTRD-1}} max\left( \theta\left( i \right) \right)-\text{min}\left( \theta\left( i \right) \right)}{\text{GSTRD-2}}$$

- Standard deviation of Gait Excursion:

$$\text{GEXC-SD}=\sqrt{\frac{\sum_{i=1}^{GTSTRD-2} \left( \text{GEXC}\left( i \right)-\text{GEXC} \right)^{2}}{\text{GTSTRD}-2}\backslash}$$

# Cognitive Responses MCDT

In this section are reported three tables encompassing the median and interquartile range concerning the total number of responses, the number of correct responses, and the number of errors given during the MCDTs by the three groups (*e.g.* OA, MCI, SCI). Notably, Table 3 concerns the ULMF tasks, namely: FTAP and THFF; Table 4 concerns the LLMF tasks, namely TTHP and HTTP; eventually, Table 5 encompasses the gold standard task for MCDT, namely: GAIT.

Table 3. The table reports the median values and the interquartile ranges (in brackets) for the three groups of subjects (OA, SCI, MCI) for the total number of responses (n responses), the number of correct responses (n correct responses), and the number of errors (n of errors) for the exercises related to ULMF (e.g., FTAP and THFF) across all three cognitive loads (CL1, CL2, CL3).

|  |  | **FTAP CL1** |  |  |  | **THFF CL1** |  |  |
| --- | --- | --- | --- | --- | --- | --- | --- | --- |
|  | **n responses** | **n correct responses** | **n of errors** |  | **n responses** | **n correct responses** | **n of errors** |  |
| **OA** | 13.5 (6) | 13 (6) | 0 (0) |  | 14.5 (8) | 14 (8) | 0 (0) |  |
| **SCI** | 15 (2.5) | 15 (2.5) | 0 (0) |  | 15 (5) | 15 (5) | 0 (0) |  |
| **MCI** | 13 (3.5) | 13 (2.5) | 0 (0) |  | 13 (5.5) | 13 (5.5) | 0 (0) |  |
|  |  | **FTAP CL2** |  |  |  | **THFF CL2** |  |  |
|  | **n responses** | **n correct responses** | **n of errors** |  | **n responses** | **n correct responses** | **n of errors** |  |
| **OA** | 8 (4) | 8 (4) | 0 (0) |  | 8.5 (3) | 8 (2) | 0 (1) |  |
| **SCI** | 9 (3) | 9 (3.25) | 0 (0.25) |  | 10 (3) | 9 (3.25) | 0 (1) |  |
| **MCI** | 7 (3.25) | 5 (4.25) | 0 (1) |  | 6 (3.25) | 5 (4.25) | 0 (1) |  |
|  |  | **FTAP CL3** |  |  |  | **THFF CL3** |  |  |
|  | **n responses** | **n correct responses** | **n of errors** |  | **n responses** | **n correct responses** | **n of errors** |  |
| **OA** | 6 (1) | 5 (3) | 0 (1) |  | 6 (3) | 5.5 (4) | 0 (1) |  |
| **SCI** | 6 (3) | 6 (3) | 0 (1) |  | 6 (1.25) | 5 (2) | 1 (1) |  |
| **MCI** | 4 (2) | 2 (1.25) | 1 (0.25) |  | 5 (1.25) | 3 (3) | 1 (1) |  |

Table 4. The table reports the median values and the interquartile ranges (in brackets) for the three groups of subjects (OA, SCI, MCI) for the total number of responses (n responses), the number of correct responses (n correct responses), and the number of errors (n of errors) for the exercises related to LLMF (e.g., TTHP and HTTP) across all three cognitive loads (CL1, CL2, CL3).

|  |  | **TTHP CL1** |  |  |  | **HTTP CL1** |  |  |
| --- | --- | --- | --- | --- | --- | --- | --- | --- |
|  | **n responses** | **n correct responses** | **n of errors** |  | **n responses** | **n correct responses** | **n of errors** |  |
| **OA** | 15 (6) | 15 (6) | 0 (0) |  | 16 (9) | 16 (9) | 0 (0) |  |
| **SCI** | 17 (4.5) | 17 (4.5) | 0 (0) |  | 15 (4.5) | 15 (4.5) | 0 (0) |  |
| **MCI** | 13 (3.5) | 13 (3.5) | 0 (0) |  | 14 (4.75) | 14 (4.75) | 0 (0) |  |
|  |  | **TTHP CL2** |  |  |  | **HTTP CL2** |  |  |
|  | **n responses** | **n correct responses** | **n of errors** |  | **n responses** | **n correct responses** | **n of errors** |  |
| **OA** | 8.5 (1) | 8 (3) | 0 (1) |  | 8 (2) | 8 (2) | 0 (1) |  |
| **SCI** | 10 (4) | 9 (4.25) | 0 (0.1) |  | 10 (3) | 10 (3) | 0 (0) |  |
| **MCI** | 6 (3.25) | 6 (3.75) | 0 (1.25) |  | 7 (4.5) | 6 (5.25) | 1 (1) |  |
|  |  | **TTHP CL3** |  |  |  | **HTTP CL3** |  |  |
|  | **n responses** | **n correct responses** | **n of errors** |  | **n responses** | **n correct responses** | **n of errors** |  |
| **OA** | 5 (2) | 4.5 (3) | 1 (1) |  | 5.5 (2) | 5.5 (3) | 0.5 (1) |  |
| **SCI** | 6 (3) | 6 (2.25) | 0 (1.25) |  | 7 (2.5) | 6 (3.25) | 0 (1) |  |
| **MCI** | 5 (1.5) | 3 (2) | 1 (1) |  | 4 (3) | 3 (2) | 1 (1.25) |  |

Table 5. The table reports the median values and the interquartile ranges (in brackets) for the three groups of subjects (OA, SCI, MCI) for the total number of responses (n responses), the number of correct responses (n correct responses), and the number of errors (n of errors) for the GAIT exercises across all three cognitive loads (CL1, CL2, CL3).

|  |  | **GAIT CL1** |  |  |
| --- | --- | --- | --- | --- |
|  | **n responses** | **n correct responses** | **n of errors** |  |
| **OA** | 10.5 (5) | 10 (4) | 0 (1) |  |
| **SCI** | 12 (4) | 12 (4) | 0 (0) |  |
| **MCI** | 11 (4.75) | 11 (4.75) | 0 (0) |  |
|  |  | **GAIT CL2** |  |  |
|  | **n responses** | **n correct responses** | **n of errors** |  |
| **OA** | 6.5 (4) | 6.5 (4) | 0 (1) |  |
| **SCI** | 8 (5) | 8 (5) | 0 (1) |  |
| **MCI** | 5 (2) | 5 (3) | 0 (1) |  |
|  |  | **GAIT CL3** |  |  |
|  | **n responses** | **n correct responses** | **n of errors** |  |
| **OA** | 4.5 (2) | 4 (3) | 0.5 (1) |  |
| **SCI** | 6 (4) | 5 (4) | 0 (1) |  |
| **MCI** | 3 (2.5) | 2 (2) | 1 (1.25) |  |

# Evaluation Metrics for Logistic Regressions Models

The evaluation metrics calculated to assess the performance of the two-classes of logistic regression models are the following:

- Sensitivity or True Positive Rate (TPR):

$$\text{TPR}=\frac{\text{True Positive}}{\text{True Positive}+\text{False Negative}}$$

- Specificity or True Negative Rate (TNR):

$$\text{TNR}=\frac{\text{True Negative}}{\text{True Negative}+\text{False Positive}}$$

- Accuracy or ACC:

$$\text{ACC}=\frac{\text{True Positive}+\text{True Negative}}{\text{True Positive}+\text{True Negative}+\text{False Positive}+\text{False Negative}}$$

The evaluation metrics concerning the three-classes models are the following:

- Recall:

$$\text{Recall}=\frac{\text{True Positive}}{\text{True Positive}+\text{False Negative}}$$

- Precision:

$$\text{Precision}=\frac{\text{True Positive}}{\text{True Positive}+\text{False Positive}}$$

- F1-Score:

$$\text{F1-Score}=\frac{2\times\text{precision}\times\text{recall}}{\text{precision}+\text{recall}}$$
